# Chromosomal evidence of species status and evolutionary relationships of the black fly *Prosimulium petrosum* (Diptera, Simuliidae) in Armenia

**DOI:** 10.3897/CompCytogen.v10i1.6551

**Published:** 2016-01-22

**Authors:** Sergey Vlasov, Maria Harutyunova, Karine Harutyunova, Peter H. Adler

**Affiliations:** 1 Department of General Biology and Bioecology, Moscow State Regional University, Radio St. 10A, Moscow 105005, Russia; 2 Institute of Molecular Biology, Yerevan, Armenia; 3 Department of Agricultural and Environmental Sciences, Clemson University, Clemson, SC 29634-0310, U.S.A.

**Keywords:** Black flies, chromosomal inversions, homosequential species, polytene chromosomes, sex chromosomes

## Abstract

The karyotype of Armenian populations of the black fly *Prosimulium petrosum* Rubtsov, 1955 was characterized and compared with that of all other chromosomally known Palearctic members of the *Prosimulium hirtipes* group. Analysis of the polytene chromosomes established that *Prosimulium petrosum* is most closely related to European populations of *Prosimulium latimucro* (Enderlein, 1925) with which it shares an identical fixed chromosomal banding sequence. Its validity as a species, separate from *Prosimulium latimucro*, is based on its unique sex-differential sections in the expanded centromere region of chromosome I, in agreement with the unique structural configuration of the hypostomal teeth of its larvae. *Prosimulium petrosum* and *Prosimulium latimucro*, therefore, are homosequential species, demonstrating the value of a combined chromosomal and morphological approach in determining species status.

## Introduction

Chromosomal rearrangements have long been considered a driving force in speciation in certain groups of organisms, based on a wealth of evidence, much of it indirect (White 1978, Rothfels 1989, Nevo 2012). If the chromosomes have played an integral role in the speciation process, individual species might be expected to carry unique signatures in their karyotype. In the dipteran family Simuliidae, the vast majority of species are chromosomally distinct from one another, even when they cannot be distinguished reliably by morphological criteria (Adler et al. 2010). Detailed banding sequences of the polytene chromosomes in the larval salivary glands of Simuliidae, consequently, have facilitated the discovery of cryptic species, provided insights into population structure and evolutionary relationships, and positioned the Simuliidae at the forefront of knowledge about the genetics of natural populations of insects (Adler and Crosskey 2015a).

The *Prosimulium hirtipes* group is a widespread Holarctic clade of the Simuliidae, consisting of 25 species in the Palearctic Region (Adler and Crosskey 2015b, Adler and Şirin 2015). Twelve of these species have been examined chromosomally, though to various degrees of precision (reviewed by Adler and Crosskey 2015a). Four species of the group occur in Armenia: *Prosimulium frontatum* Terteryan, 1956, *Prosimulium petrosum* Rubtsov, 1955, *Prosimulium rachiliense* Djafarov, 1954, and *Prosimulium tomosvaryi* (Enderlein, 1921) (Adler and Crosskey 2015b). Analyses of the polytene chromosomes of *Prosimulium frontatum*, *Prosimulium rachiliense*, and *Prosimulium tomosvaryi* have revealed cryptic biodiversity and provided hypotheses of their phylogenetic relationships (Adler and Şirin 2014). Comparative chromosomal studies of Armenian populations of *Prosimulium petrosum*, however, are lacking, although general karyotypic features, putatively of this species, have been presented for Bulgarian populations (Ralcheva and Dryanovska 1973, Ralcheva 1974, Chubareva and Petrova 2003).

*Prosimulium petrosum* was described from larvae and pupae collected on 26 May 1952 in Azerbaijan; the holotype larva is from River Agsu above Lake Göygöl (= Geigel) (Rubtsov 1955). Adults attributed to this species were described from Azizbekov (= Vayk) in Armenia (Rubtsov 1956). Terteryan (1968), however, suggested that the descriptions of the Armenian adults represent *Prosimulium pronevitshae* Rubtsov, 1955, now a synonym of *Prosimulium rachiliense* (Crosskey and Zwick 2007, Adler and Şirin 2014). The pupal gill figured by Rubtsov (1956), based on Azerbaijanian material, has a branching formula of (2+2+2+2)+(2+2)+(2+2), whereas that by Terteryan (1968), based on Armenian material, has a formula of (3+3+2)+(2+2)+(2+2).

Given the lack of chromosomal information for bona fide material of *Prosimulium petrosum*, we conducted a comparative band-by-band analysis of *Prosimulium petrosum* to characterize its karyotype and illuminate its taxonomic status and evolutionary relationships. In particular, we were interested in determining if *Prosimulium petrosum* is a species distinct from the morphologically similar European species, *Prosimulium latimucro* (Enderlein, 1925), or if they are conspecific.

## Methods

Larvae were collected from three streams, up to about 210 km apart, in April and May in northern and southern Armenia (Table 1). The material was fixed in a 3:1 mixture of ethanol and glacial acetic acid. Pupae and adults were not collected, but 13 mature larvae with well-developed gill histoblasts were obtained. Larvae were identified morphologically as *Prosimulium petrosum*, based on structural characters (Rubtsov 1956, Terteryan 1968)—the apex of the median hypostomal tooth of our material was posterior to the apices of the lateral teeth, and the 16 filaments of the pupal gill were arranged on three, widely splayed primary trunks, with a branching formula of (3+3+2)+(2+2)+(2+2) or (3+3+2)+(2+1+1)+(2+2).

**Table 1.** Collection data for larvae of *Prosimulium petrosum* in Armenia.

Site	Location	Latitude Longitude	Altitude (m asl)	Date	Larvae analyzed males:females
1	Armenia, Gegarkunik Province, Ddmashen^†^	40°34'N 44°49'E	ca. 1900	21 April 2010	3:5
2	Armenia, Sjunik Province, Mogralzani-Vardanidzor, Megraget River	39°00.40'N 46°12.45'E	ca. 1265	04 May 2011	0:1
3	Armenia, Sjunik Province, Megrinsky pass	39°06.30'N 46°10.47'E	ca. 2375	04 May 2011	13:41

† Exact location in Ddmashen area is unknown.

**Figure 1.**
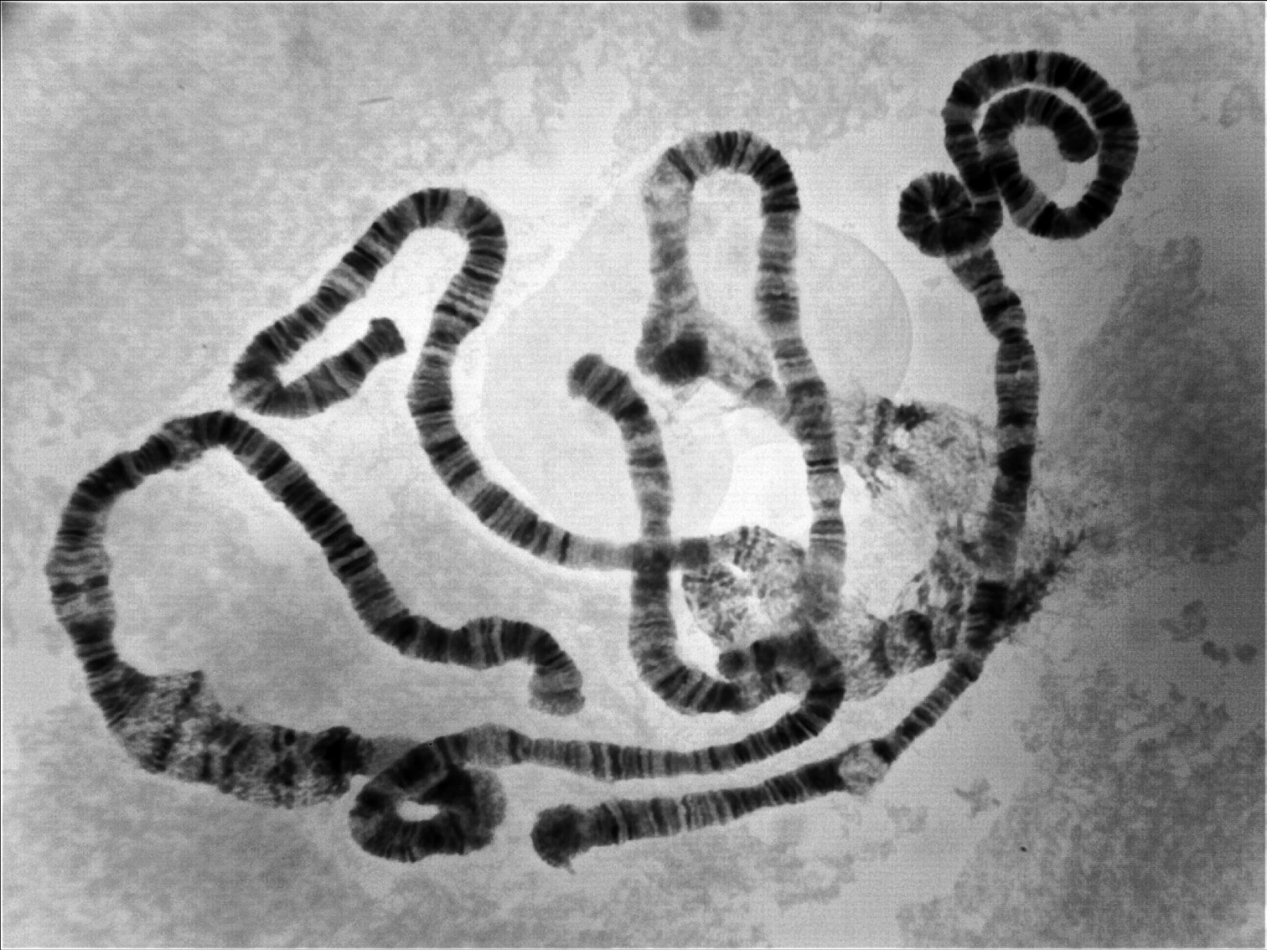
Total polytene chromosomal complement of *Prosimulium petrosum*. Total polytene chromosomal complement of female larva of *Prosimulium petrosum*, showing the diploid condition of 2n = 6, with tightly paired homologues.

**Figure 2.**
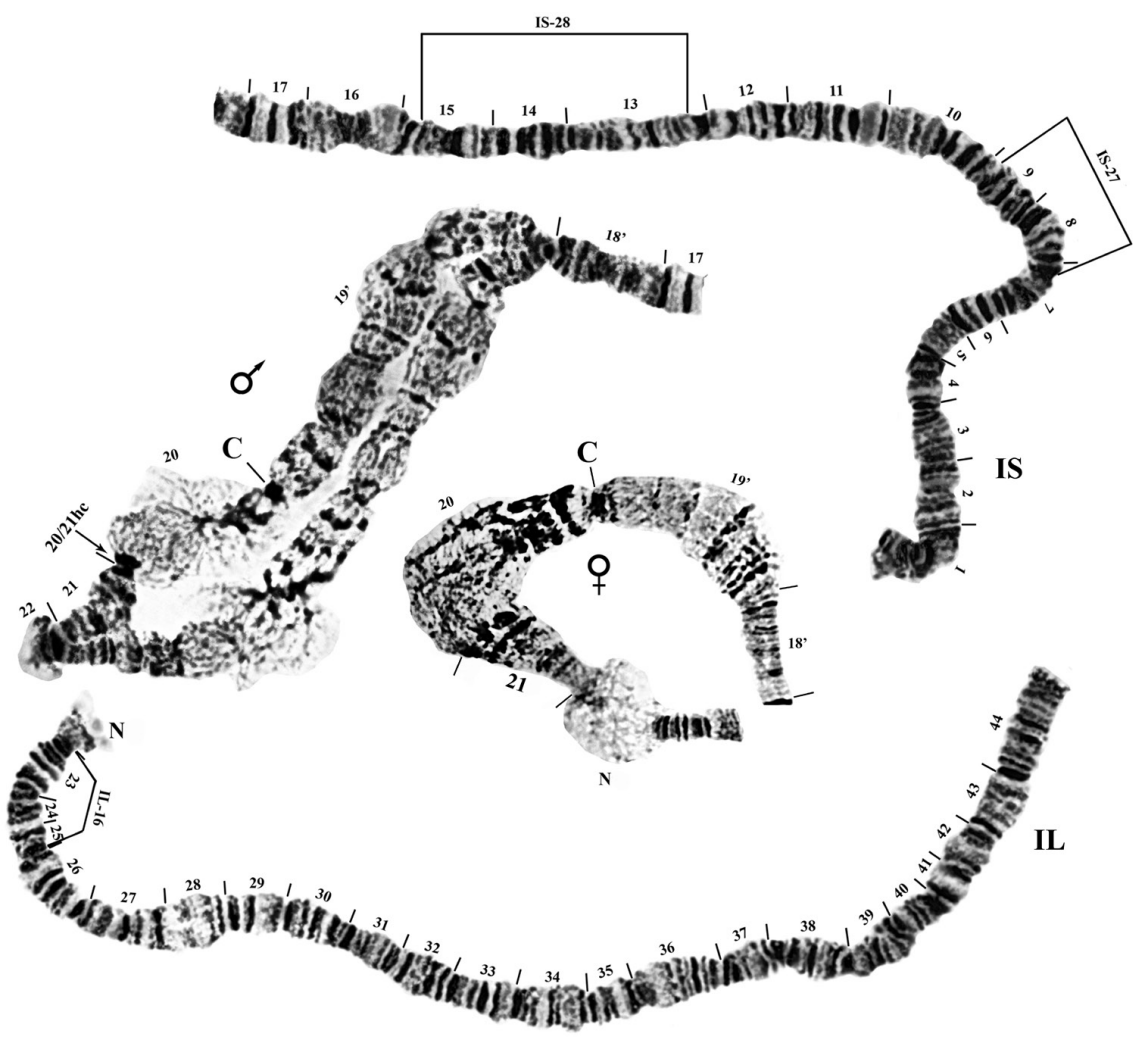
Chromosome I of *Prosimulium petrosum*, with male and female transformed centromere regions (CI_t_). Breakpoints of autosomal heterozygous inversions are indicated by brackets. C: centromere, NO: nucleolar organizer, 20/21hc: heterochromatic band.

**Figure 3.**
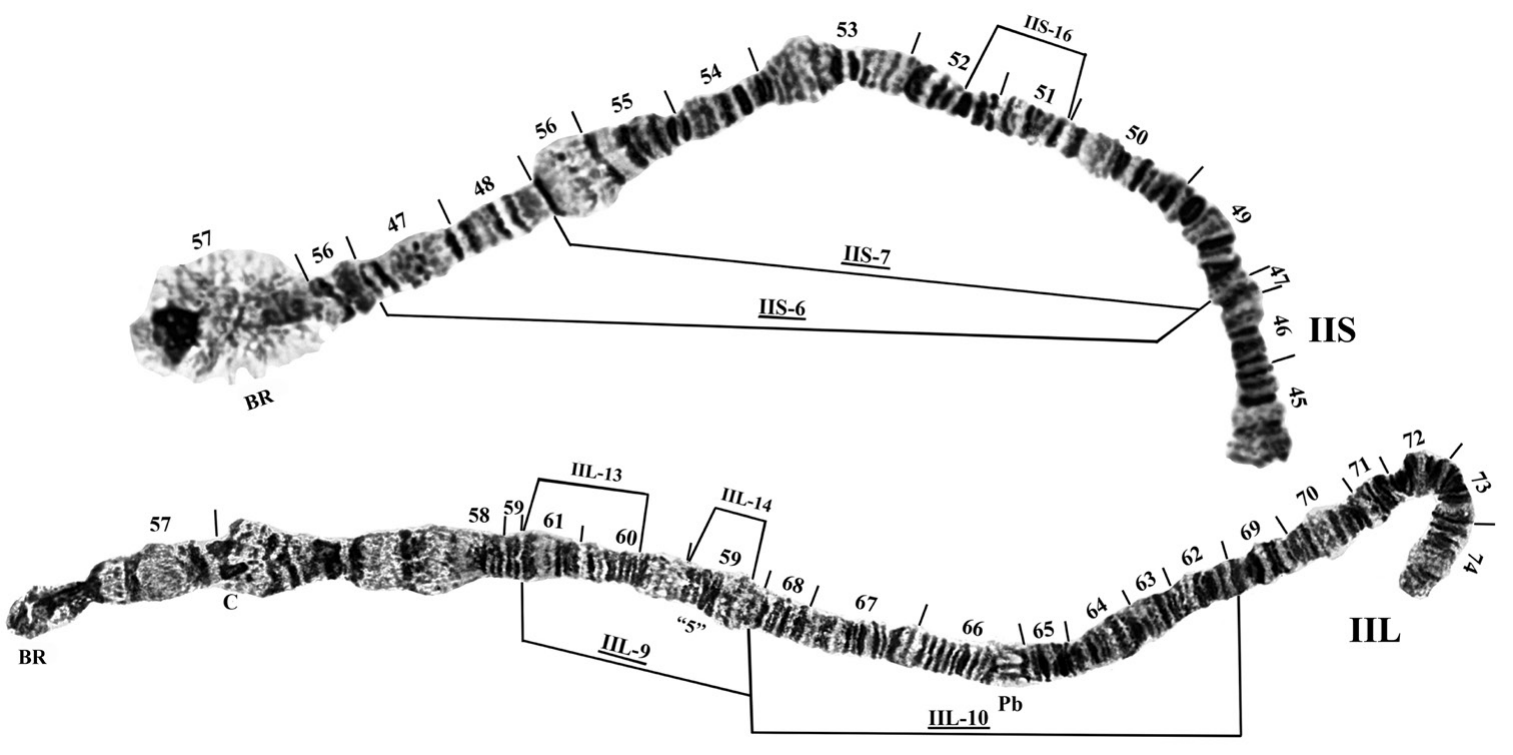
Chromosome II of *Prosimulium petrosum*. Relative to the standard sequence, fixed inversions *IIS-6*, *IIS-7*, *IIL-9*, and *IIL-10* are present. Breakpoints of autosomal inversions are indicated by brackets above the chromosomes. BR: Balbiani ring, C: centromere, Pb: parabalbiani, “5”: group of 5 marker.

**Figure 4.**
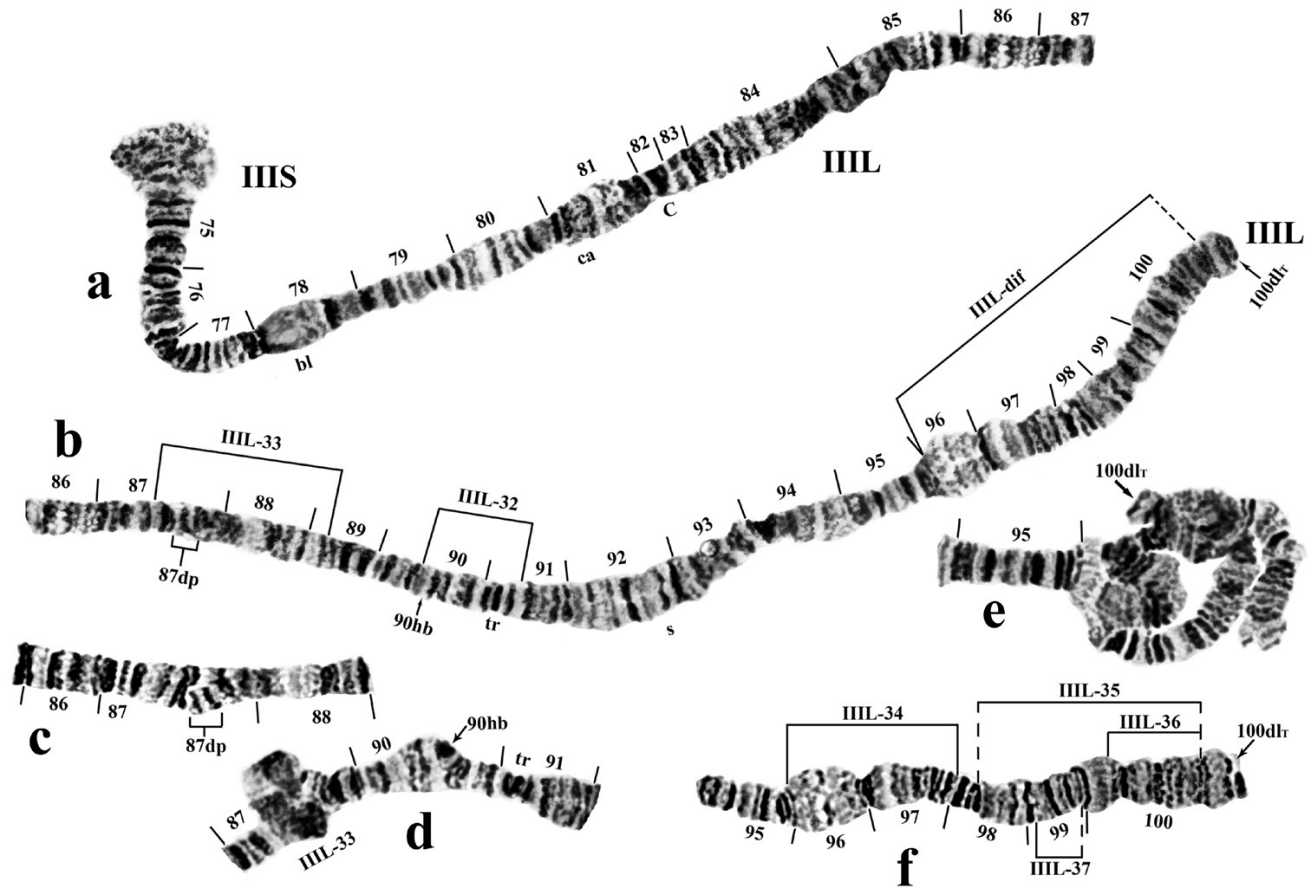
Chromosome III of *Prosimulium petrosum*. **a, b** chromosome III of *Prosimulium petrosum*. Breakpoints of autosomal inversions and location of 2 additional bands (87dp) are indicated by brackets. IIIL-dif is an inversion complex, hypothesized to consist of four inversions. Arrows indicate locations of 90hb and 100dlT **c** heterozygous band duplication 87dp **d** heteroband 90hb and heterozygous inversion IIIL-33 **e** complex set of heterozygous inversions, collectively referred to as IIIL-dif; arrow shows deletion 100dlT in telomere of one homologue. f - heterozygous deletion 100dlT; breakpoints of individual inversions IIIL-34, IIIL-35+IIIL-36+IIIL-37, which comprise complex inversion IIIL-dif, are indicated by brackets; dashed line designates approximate limits of inversions. C: centromere, bl: blister, ca: capsule, s: shield, tr: triad.

Polytene chromosomes from the larval salivary glands were stained using the lacto-aceto orcein method (Bedo 1975), which also stained gonadal tissue. Preparations were spread by squashing chromosomes on a microscope slide. Larval gender was determined by the form of the gonads: rounded in males and elongated in females. Representative chromosomal arms and selected rearrangements were photographed under oil immersion (Figs 1–4). Composite digital images from different focal planes were made with Helicon Focus 5.3 and further processed with Adobe Photoshop CS6. The banding sequences of all six chromosomal arms were compared with the standard maps of the *Prosimulium hirtipes* group (Basrur 1962) and with maps of various species in the *Prosimulium hirtipes* group (Basrur 1959, Adler and Belqat 2001, Adler and Şirin 2014).

Fixed inversions (i.e., homozygous in all larvae) are italicized in the text and underlined on our maps; floating inversions (i.e., polymorphisms) are not italicized or underlined. Inversions identical to those identified in previous studies (i.e., *IIS-6*, *IIS-7*, *IIL-9*, and *IIL-10*) were given the same numbers assigned by Basrur (1959). Newly discovered inversions were numbered to follow the last number assigned to inversions in other species of the *Prosimulium hirtipes* group currently under study and as yet unpublished. Chromosomal terminology, including terms for landmarks, follows that of Basrur (1959, 1962).

Three morphological preparations of mature larvae were deposited in the Zoological Institute of the Russian Academy of Sciences, St. Petersburg, Russia. Additional morphological preparations and chromosomal photographs were deposited in the Moscow State Regional University, Moscow, Russia.

## Results

**Karyotype.** In total, 64 larvae were analyzed. One larva from Site 1 chromosomally matched the banding sequence of *Prosimulium rachiliense* cytoform ‘A’ (*sensu* Adler and Şirin 2014). The other 63 larvae (16 males, 47 females) were assigned to *Prosimulium petrosum*. All larvae had a diploid number of 2n = 6, with tightly paired homologues (Fig. 1).

The chromosomes were submetacentric (Fig. 1). Chromosome I (sections 1–44) was the longest, with the two arms (IS and IL) subequal in length, followed by chromosome II (sections 45–74) with the long arm (IIL) slightly longer than the short arm (IIS). Chromosome III (sections 75–100) was the shortest, with the long arm (IIIL) approximately 35% longer than the short arm (IIIS). The centromere regions of chromosomes I and II were transformed (CI_t_, CII_t_; *sensu* Basrur 1959), producing an expanded, flocculent area from section 19 through section 21 (CI_t_) and from the middle of section 57 through section 58 (CII_t_) (Figs 1–3). The centromere region of chromosome III was not expanded (Figs 1, 4).

A single, primary nucleolus organizer was in the standard position for the *Prosimulium hirtipes* group, that is, in the base of IL (Fig. 2). Landmarks that remained in the standard positions for the *Prosimulium hirtipes* group included the single Balbiani ring in the base of IIS (Fig. 3), “blister” in IIIS (Fig. 4), and “shield” and “triad” in IIIL (Fig. 4). A chromocenter was lacking, and supernumerary (B) chromosomes were absent.

**Fixed (interspecific) inversions.** The banding sequence of chromosome arms IS, IL, IIIS, and IIIL was identical with the standard banding sequence established by Basrur (1959, 1962) for the *Prosimulium hirtipes* group. Chromosome II, however, had four fixed inversions relative to the standard sequence—two overlapping inversions in the short arm, *IIS-6* and *IIS-7*, and two tandem inversions in the long arm, *IIL-9* and *IIL-10* (Fig. 3). The four inversions involved 73% and 58% of the sections of IIS and IIL, respectively. *IIL-9* moved the “group of 5” marker more centrally and *IIL-10* reversed the polarity of the parabalbiani.

**Autosomal (intraspecific) polymorphisms.** Fifteen autosomal polymorphisms were detected; all were present in the heterozygous state only. These autosomal rearrangements included 12 inversions, one heteroband (90hb), a duplication of bands (87dp), and a telomeric deletion (100dlT) (Table 2, Figs 2, 3, 4). Four inversions in sections 96–100 of IIIL formed a complex set of loops collectively referred to as IIIL-dif (Fig. 4, e). The proposed breakpoints of these four inversions are shown in Fig. 4, f. All autosomal polymorphisms were expressed in low frequency (< 0.065; Table 2).

**Table 2.** Frequency of homologues with autosomal inversions and other rearrangements (band deletions, duplications, and heterobands) in three Armenian populations of *Prosimulium petrosum*.

Collection site	1	2	3	Armenia^†^
Larvae (*n*)	8	1	54	63
Chromosomal homologues (*n*)*	16	2	108	126
IS-27	0.063		0.037	0.040
IS-28	0.063			0.008
IL-16	0.063		0.009	0.016
IIS-16			0.009	0.008
IIL-13			0.009	0.008
IIL-14			0.028	0.024
IIIL-32			0.019	0.016
IIIL-33			0.028	0.024
IIIL-dif^‡^			0.028	0.024
100dlT^‡^			0.046	0.040
90hb			0.019	0.016
87dp			0.009	0.008
Mean number of heterozygous inversions/larva^§^				0.333
Mean number of all heterozygous chromosomal rearrangements/larva^§^				0.460

† All three collection sites combined. * Frequencies of rearrangements were calculated on the basis of the number of homologues.

‡ Three larvae, which had IIIL-dif (= IIIL-34, IIIL-35+IIIL-36+IIIL-37), also carried heterozygous deletion 100dl_T_, the frequency of which is accounted for separately; two additional larvae had heterozygous deletion 100dl_T_ in the absence of IIIL-dif.

§ IIIL-dif was treated as a single inversion for the purpose of presenting means.

**Sex-differential region.** All 16 males had a heterochromatic band (20/21hc) at the junction of sections 20 and 21 and lacked conjugation in the CI_t_ region, typically from section 20 through the beginning of section 21 (Fig. 2), although one male was unpaired from the beginning of section 19 to the beginning of section 21; no inversion could be discerned in the unpaired region. Females lacked the heterochromatic band and exhibited complete pairing of homologues in the CI_t_ region. Thus, the expanded centromere region of chromosome I was the sex-differential segment, with males X_0_Y_1_ and females X_0_X_0_. In two males, ectopic pairing of CI_t_ and CII_t_ occurred in some nuclei.

## Discussion

Our chromosomal analysis requires taxonomic context, especially a reasonable assignment of the correct species name. The larvae from our three sites in Armenia are chromosomally cohesive. Based on gill structure, they conform to previous Armenian collections (Terteryan 1968), rather than to Azerbaijanian material (Rubtsov 1956), of *Prosimulium petrosum*. Based on hypostomal structure, they precisely match the Azerbaijanian (holotype) (Rubtsov 1956). Our Armenian collections and the type locality of *Prosimulium petrosum* in Azerbaijan are 140–160 km apart, and all are in the same ecoregion—the Caucasus Mixed Forests Ecoregion (World Wildlife Federation 2015). Although a slight difference in the branching pattern of the gill between Armenian and Azerbaijanian samples possibly indicates the presence of cryptic species, we attribute the difference to intraspecific variation, which is common, especially on the dorsal trunk, in members of the *Prosimulium hirtipes* group (Stloukalova 2004). Similarly, although we found mature larvae 1.0–1.5 months earlier (end of April–beginning of May) than did Terteryan (1968), the seasonal difference could be attributable to altitude or perhaps climatic variation among years. Given the minimal geographic distance, dispersal ability of simuliids (Adler et al. 2005), identical ecoregion, and morphological similarity, we conclude that our Armenian populations are conspecific with the holotype.

Although our material corresponds with the type (Caucasican) concept of *Prosimulium petrosum* (Rubtsov 1955), a larger question is whether *Prosimulium petrosum* is a unique species or conspecific with *Prosimulium latimucro*, as suggested by Adler and Crosskey (2015b), based on morphological similarity. Yankovsky (2003) suggested that the projection of the median hypostomal tooth anterior to the lateral teeth and the second-order branching (i.e., 3+3+2) of the upper gill trunk distinguish the larva of *Prosimulium latimucro* from that of *Prosimulium petrosum*. Accordingly, our samples correspond with *Prosimulium petrosum*, based on the hypostomal teeth, and with *Prosimulium latimucro*, based on the branching of the gill.

What do the banding sequences of the polytene chromosomes reveal about possible conspecificity of *Prosimulium petrosum* and *Prosimulium latimucro* and their evolutionary relationships? The Armenian population of *Prosimulium petrosum* shares *IIS-6,7* and *IIL-9* with *Prosimulium latimucro*, *Prosimulium rufipes* (Meigen, 1830), and *Prosimulium* “aff. 3” of Basrur (1959), and fixation of *IIL-10* with *Prosimulium rufipes*, *Prosimulium* “aff. 3”, and Moroccan *Prosimulium latimucro* (Adler and Belqat 2001, Adler and Şirin 2014). In European populations of *Prosimulium latimucro*, inversion IIL-10 is absent or polymorphic. *Prosimulium petrosum* differs from *Prosimulium rufipes*, *Prosimulium* “aff. 3”, and Moroccan *Prosimulium latimucro* by lacking *IS-18*, *IIS-8*, and *IIL-11*, respectively. *Prosimulium petrosum* does not share any autosomal polymorphisms with any studied member of the *Prosimulium hirtipes* group. We conclude that European populations of *Prosimulium latimucro* are most closely related to *Prosimulium petrosum*.

Males and females of *Prosimulium petrosum* consistently differ in the expression of their CI_t_ region, indicating the general location of the sex-determining locus. The sex chromosomes of the Simuliidae often are associated with rearrangements, such as inversions and heterobands, although the X and Y also can be microscopically undifferentiated (X_0_Y_0_) (Rothfels 1980, Post 1982). Any of the three chromosomes (I, II, or III) can function as the sex chromosome. Identical, differentiated sex chromosomes are rarely shared between species (Rothfels and Freeman 1983, Adler et al. 2015). Thus, the sex chromosomes can be useful in species discovery and identification (Rothfels 1989).

Lack of pairing of homologues in the CI_t_ region, observed in males of *Prosimulium petrosum*, also is found in at least some populations of other Palearctic members of the *Prosimulium hirtipes* group, such as *Prosimulium hirtipes* (Fries, 1824), *Prosimulium latimucro*, and *Prosimulium* “aff. 3”, and often serves as the basis for further elaboration of the Y chromosome, such as the addition of sex-linked inversions and heterobands (Basrur 1959, Adler unpublished). The heterochromatic band 20/21hc of *Prosimulium petrosum* also appears on the Y chromosome of various members of the *Prosimulium hirtipes* group, including Moroccan populations of *Prosimulium rufipes* and *Prosimulium latimucro* and some European populations of *Prosimulium latimucro* and *Prosimulium* “aff. 3”, often with various repatternings of banding in the CI_t_ region (Adler and Belqat 2001, Adler unpublished). No conspicuous repatterning was observed in the CI_t_ region of *Prosimulium petrosum*. We do not know if the unpaired CI_t_ condition and heterochromatic band 20/21hc are identical across populations and species, and if so, if their shared nature reflects common ancestry, introgression, or independent origins. Species differences in other members of the *Prosimulium hirtipes* group, such as those in eastern North America often are based on minor differences in the centromere region, especially of CIII (Rothfels and Freeman 1977).

A Y chromosome based on an unpaired CI_t_ region, coupled with 20/21hc, without an associated inversion or band repatterning, uniquely characterizes *Prosimulium petrosum*. The allopatric nature of *Prosimulium petrosum* and *Prosimulium latimucro*, however, presents a challenge for evaluating reproductive isolation; the nearest chromosomally analyzed populations of *Prosimulium petrosum* and *Prosimulium latimucro* are more than 1,500 km apart. Our analysis of the photographs by Ralcheva (1974) of putative *Prosimulium petrosum* from Bulgaria suggests that IS and IL are standard, *IIS-6,7* and *IIL-9* are present, and IIL-10 is absent; the sex chromosomes and larval morphology were not mentioned. Based on available evidence, *Prosimulium petrosum* of Ralcheva (1974), therefore, is probably *Prosimulium latimucro*, and most closely resembles populations in the Swiss Alps (as *Prosimulium inflatum* “aff. 1” of Basrur 1959). The chromosomal characteristics of all other analyzed populations identified as *Prosimulium petrosum* and *Prosimulium latimucro* are entirely congruent with the respective configurations of the hypostomal teeth. Thus, we argue that *Prosimulium petrosum* is a distinct species on the basis of unique chromosomal features corroborated by distinct hypostomal features. *Prosimulium petrosum* and European *Prosimulium latimucro*, therefore, are homosequential species—they have the same fixed banding sequence but differ morphologically, a phenomenon first discovered in *Drosophila* (Carson et al. 1967). Although not common in the Simuliidae, previous examples of homosequential species include several members of the *Simulium vernum* group (Hunter 1987, Adler et al. 2004, Seitz and Adler 2015).
